# A synchrotron study of Na_2.27_Ho_7.73_(SiO_4_)_6_O_0.72_

**DOI:** 10.1107/S1600536809011866

**Published:** 2009-04-08

**Authors:** Ivonne Rosales, Eligio Orozco, Lauro Bucio, Maria E. Fuentes, Luis Fuentes

**Affiliations:** a Instituto de Física, Universidad Nacional Autónoma de México, AP 20-364, 01000 México DF, Mexico; b Facultad de Química, Universidad Autónoma de Chihuahua, Chihuahua, Mexico; c Centro de Investigación en Materiales Avanzados, Chihuahua, Mexico

## Abstract

A well crystallized powder sample of sodium holmium orthosilicate oxyapatite, Na_2.27_Ho_7.73_(SiO_4_)_6_O_0.72_, was obtained after mechanical milling and thermal treatment at 1123 K. Crystal structure analysis was performed from the results of Rietveld refinement of the synchrotron diffraction data. As in other rare-earth orthosilicate apatites, sodium cations appear located sharing with holmium the 4*f* Wyckoff position at the center of a tricapped trigonal prism. In its turn, holmium almost fully occupies the 6*h* position at the center of a seven-coordinated penta­gonal bipyramid. A small quantity of Na atoms was found at this site. No vacancies are present in the two independent crystallographic sites available for Ho and Na atoms.

## Related literature

The method of preparation was based on Rodríguez-Reyna *et al.* (2006 ▶) and Fuentes *et al.* (2006 ▶). For related structures, see: Gunawardane *et al.* (1982 ▶); Gualtieri (2000 ▶); Emirdag-Eanes *et al.* (2004 ▶); Redhammer & Roth (2003 ▶). For bond-valence parameters for oxides, see: Brese & O’Keeffe (1991).

## Experimental

### 

#### Crystal data

Ho_7.73_Na_2.27_O_24.72_Si_6_
*M* *_r_* = 1891.09
Hexagonal, 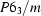

*a* = 9.3405 (1) Å
*c* = 6.7638 (1) Å
*V* = 511.05 (1) Å^3^
*Z* = 1
Synchrotron radiation
λ = 1.033490 (7) Å
*T* = 295 K
Specimen shape: flat sheet
15 × 15 × 0.2 mm
Specimen prepared at 1123 K

#### Data collection

SSRL diffractometer
Specimen mounting: packed powder sample container
Specimen mounted in reflection mode
Scan method: step
2θ_min_ = 5, 2θ_max_ = 70.0°
Increment in 2θ = 0.01°

#### Refinement

*R*_p_ = 0.10
*R*_wp_ = 0.14
*R*_exp_ = 0.11
*R*_B_ = 0.04
*S* = 1.27
Excluded region(s): none
Profile function: conventional pseudo-Voigt
281 reflections
22 parameters
Preferred orientation correction: none

### 

Data collection: *SSRL Software*; cell refinement: *DICVOL* (Boultif & Louër, 2004 ▶); data reduction: *FULLPROF* (Rodríguez-Carvajal, 2006 ▶); method used to solve structure: coordinates taken from an isotypic compound; program(s) used to refine structure: *FULLPROF*; molecular graphics: *ATOMS* (Dowty, 1994 ▶); software used to prepare material for publication: *ATOMS*.

## Supplementary Material

Crystal structure: contains datablocks global, I. DOI: 10.1107/S1600536809011866/br2094sup1.cif

Rietveld powder data: contains datablocks I. DOI: 10.1107/S1600536809011866/br2094Isup2.rtv

Additional supplementary materials:  crystallographic information; 3D view; checkCIF report

## Figures and Tables

**Fig. 1.**
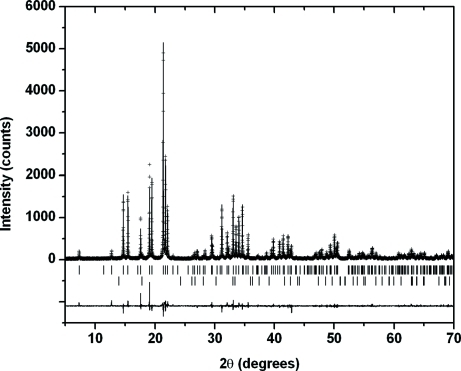
Rietveld refinement for OAp employing synchrotron radiation data. Observed (crosses), calculated (solid line) and difference (bottom trace) plots are represented. Vertical marks correspond to the allowed Bragg reflections for OAp (first row) and SiO2 (second row).

**Fig. 2.**
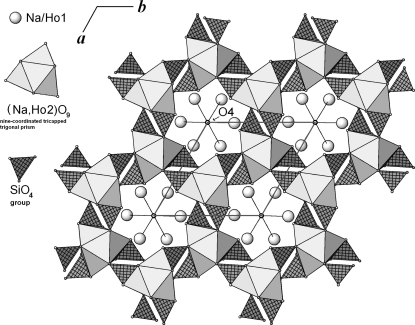
, Structural representation of Na2.27Ho7.73(SiO4)6O0.72.

## supplementary crystallographic information

### Comment

Apatites with general formula *M*_10_(XO_4_)_6_*Z*_2_ commonly have cationic and anionic substitutions generating different structural arrangements. In the preceding chemical formula, *M* is a divalent atom (typically Ca^2+^ and others); *Z* represents F^-^, OH^-^, Br^-^, Cl^-^ or O^2-^; while *X* frequently is P^5+^ and in some cases Si^4+^, Ge^4+^ or V^4+^. Most apatites crystallize in the hexagonal system with symmetry given by the space group P6_3_/*m* (No.176). Charge balance is assured by the presence of point defects or cationic vacancies in cases in which the element *M* splits in *M*' and *M*'' being *M*' an alkaline monovalent ion and *M*'' a trivalent metal giving rise to *M*'*_x_*M''_10 - _*_x_*(SiO_4_)_6_O_3 - _*_x_* oxyapatites, as in NaNd_9_(GeO_4_)_6_O_2_ (Emirdag-Eanes *et al.*, 2004) and LiY_9_(SiO_4_)_6_O_2_ (Redhammer & Roth, 2003). The structural data used for the title OAp phase was established considering the isostructural compound NaY_9_(SiO_4_)_6_O_2_ reported by Gunawardane *et al.* (1982). For this compound, the chemical formula Na*_x_*Y_10 - _*_x_*(SiO_4_)_6_O_2_ represents a model in which the sodium ion is introduced in the apatite structure substituting for yttrium. In the present work the nominal formula used for holmium oxyapatite and the possible incorporation of sodium and fluorine is represented as Na*_x_*Ho_10 - _*_x_*(SiO_4_)_6_O_2 - _*_y_*F*_y_*. The crystal structure of OAp has an arrangement similar to some other alkaline rare-earth oxyapatites already reported such as NaY_9_(SiO_4_)_6_O_2_ and LiY_9_(SiO_4_)_6_O_2_ (Redhammer & Roth, 2003).

### Experimental

The mechanical milling at 612 rpm (FRITSCH Pulverisette mill model 06.102) of sodium holmium orthosilicate oxyapatite (OAp) was carried out considering the nominal composition Na_2_Ho_8_(SiO_4_)_6_F_2_ from stoichiometric mixtures of Ho_2_O_3_ (Aldrich.99.9%), SiO_2_ (Aldrich 99.6%) and NaF (Analit.Analytic grade) following the method of Rodríguez-Reyna *et al.* (2006) and Fuentes *et al.* (2006). Once the mechanochemical processing finished, the sample was heated in a tube furnace for 15 h at 1073 K in air atmosphere. Conventional X-ray powder diffraction data showed reflections that match with the isostructural oxyapatite NaY_9_(SiO_4_)_6_O_2_ (PDF file 35–404). At this step of synthesis, the sample exhibited very poor crystallinity. After an additional thermal treatment at 1123 K, the crystallization of the sample was reached with the presence of quartz (PDF file 46–1045) as a secondary phase. From wavelength dispersive spectrometry (WDS),chemical analysis was performed by means of a Jeol JXA-8900*R*, EPMA spectrometer.

The average atomic content with standard uncertainty for each element in the sample were 12.4(1.9) for sodium, 42.2(4.6) for holmium and 45.2(6.3) for silicon, and give a Na:Ho ratio of 0.29. The expected values, according to the initial mixture of reactives were 12.5 for sodium, 50.0 for holmium and 37.5 for silicon. These quantities changed as consequence of the process of mechanical milling. Fluorine could not be observed so the phase originally labeled as OAp could be considered as pure oxyapatite phase. The atomic content for each element in the unit cell for OAp was calculated dividing by 5.46 the averaged WDS values giving 2.27 for sodium, 7.23 for holmium and 8.28 for silicon. The excess of silicon was interpreted taking into account the presence of quartz measured as a secundary phase by *x*-ray diffraction. Therefore, the content of silicon had to be split in such way that 6 silicon atoms were corresponded to OAp and 2.28 to quartz for each unit cell of oxyapatite.

### Refinement

The incident beam was calibrated using LaB_6_ (*NIST* SRM 660) as reference standard. The starting parameters for performing the Rietveld refinement were, for the OAp phase, the data from the isostructural NaY_9_(SiO_4_)_6_O_2_ (ICSD 27191, Gunawardane *et al.*, 1982); and for quartz the data from ICSD 90145 (Gualtieri, 2000). Atomic coordinates were refined for the OAp phase considering the holmium and sodium atoms distributed in each one of the two available sites according to the Na:Ho ratio of 0.29 (obtained by WDS). In the following step, the occupation factors for Na and Ho were constrained in such a way that the total amount of both Na and Ho be constant keeping the Na:Ho ratio at 0.29. This ratio was allowed to change for each one of the crystallographic sites. At the end of the refinement, the thermal isotropic parameters were refined independently for each site. These parameters were set to the same refined value for all the O atoms in the structure.

Bond valence calculations were made using the recommended bond-valence parameters for oxides published by Brese & O'Keeffe (1991). Bond valence sum around Ho1 and Ho2 gave the values of 3.01 and 2.75 respectively, being the last lower than the expecting valence of 3. One way to explain the low bond valence sum is considering the Na substituting for Ho. In such case, bond valence sum for Na2 in the Ho2 site is found to be 1.51, and the weighted bond valence sum results equal to the weighted atomic valence when the fraction of Na2 is 0.33 in the Ho2 site. This quantity is reasonably close to 0.38 obtained by Rietveld refinement. For the Ho1 site, the bond valence sum around Ho is practically the expecting valence of 3 which agrees with the very low proportion of Na (0.12) obtained in the refinement. For the Ho1—O4 bonds there are three Ho1 atoms coordinated to O4 oxygen with a bondlengths of 2.197 Å. Each Ho1—O4 bond contributes with a bond valence of 0.62 giving a bond valence sum of 1.87. This value agrees with the fact that this site is partialy occupied by oxygen O4 by an occupation factor of 0.36. With all this results, the chemical formula obtained was found to be Na_2.27_Ho_7.73_(SiO_4_)_6_O_0.72_, which is charge balanced. The composition agrees with the WDS results and sodium content. The final Rietveld refinement of the synchrotron diffraction pattern is shown in Fig. 1 and the crystal structure is represented in Fig. 2.

### Crystal data

**Table:**

Ho_7.73_Na_2.27_O_24.72_Si_6_	*F*(000) = 822.8
*M**_r_* = 1891.09	*D*_x_ = 6.145 Mg m^−^^3^
Hexagonal, *P*6_3_/*m*	Synchrotron radiation, λ = 1.033490 Å
Hall symbol: -P 6c	*T* = 295 K
*a* = 9.3405 (1) Å	white
*c* = 6.7638 (1) Å	flat sheet, 15 × 15 mm
*V* = 511.05 (1) Å^3^	Specimen preparation: Prepared at 1123 K
*Z* = 1	

### Data collection

**Table:**

SSRL diffractometer	Data collection mode: reflection
Radiation source: synchrotron source	Scan method: step
Si(111)	2θ_min_ = 5.0°, 2θ_max_ = 70.0°, 2θ_step_ = 0.01°
Specimen mounting: packed powder sample container	

### Refinement

**Table:**

Least-squares matrix: full with fixed elements per cycle	Excluded region(s): none
*R*_p_ = 0.10	Profile function: conventional pseudo-Voigt
*R*_wp_ = 0.14	22 parameters
*R*_exp_ = 0.11	0 restraints
*R*_Bragg_ = 0.04	Weighting scheme based on measured s.u.'s
*R*(*F*^2^) = 0.03	(Δ/σ)_max_ = 0.05
χ^2^ = 1.613	Background function: linear interpolation between a set of background points with refinable heights
6501 data points	Preferred orientation correction: none

### Fractional atomic coordinates and isotropic or equivalent isotropic displacement parameters (Å^2^)

**Table:**

	*x*	*y*	*z*	*U*_iso_*/*U*_eq_	Occ. (<1)
Ho1	0.2373 (2)	0.0042 (3)	0.25	0.0014 (4)	0.875 (2)
Na1	0.2373 (2)	0.0042 (3)	0.25	0.0014 (4)	0.125 (2)
Ho2	0.3333	0.6666	−0.0028 (9)	0.0013 (8)	0.620 (2)
Na2	0.3333	0.6666	−0.0028 (9)	0.0013 (8)	0.380 (2)
Si	0.368 (1)	0.397 (1)	0.25	0.014 (3)	
O1	0.246 (1)	0.334 (1)	0.444 (2)	0.030 (4)	
O2	0.490 (2)	0.312 (2)	0.25	0.030 (4)	
O3	0.523 (2)	0.403 (2)	0.75	0.030 (4)	
O4	0.0	0.0	0.25	0.030 (4)	0.358

### Geometric parameters (Å, °)

**Table:**

Ho1—O1^i^	2.42 (1)	Ho2—O2^viii^	2.29 (1)
Ho1—O1^ii^	2.24 (1)	Ho2—O2^ix^	2.29 (1)
Ho1—O1^iii^	2.42 (1)	Ho2—O2^x^	2.29 (2)
Ho1—O1^iv^	2.24 (1)	Ho2—O3^viii^	2.45 (1)
Ho1—O2	2.66 (1)	Ho2—O3^ix^	2.45 (2)
Ho1—O3^ii^	2.35 (2)	Ho2—O3^x^	2.45 (1)
Ho1—O4	2.197 (2)	Si—O1	1.64 (1)
Ho2—O1^v^	2.82 (2)	Si—O1^vi^	1.64 (1)
Ho2—O1^vi^	2.82 (1)	Si—O2	1.68 (3)
Ho2—O1^vii^	2.82 (1)	Si—O3^ix^	1.62 (2)
			
O1^v^—Ho2—O3^viii^	60.3 (7)		

Symmetry codes: (i) −*x*+*y*, −*x*, *z*; (ii) *y*, −*x*+*y*, *z*−1/2; (iii) −*x*+*y*, −*x*, −*z*+1/2; (iv) *y*, −*x*+*y*, −*z*+1;  (v) −*x*+*y*, −*x*+1, −*z*+1/2; (vi) *x*, *y*, −*z*+1/2; (vii) −*y*+1, *x*−*y*+1, −*z*+1/2; (viii) *x*−*y*, *x*, *z*−1/2; (ix) −*x*+1, −*y*+1, *z*−1/2; (x) *y*, −*x*+*y*+1, *z*−1/2.
